# Development and validation of a rapid LC–MS/MS method for the confirmatory analysis of the bound residues of eight nitrofuran drugs in meat using microwave reaction

**DOI:** 10.1007/s00216-021-03763-0

**Published:** 2021-11-23

**Authors:** Gemma Regan, Mary Moloney, Melissa Di Rocco, Padraig McLoughlin, Wesley Smyth, Steven Crooks, Christopher Elliott, Martin Danaher

**Affiliations:** 1 Institute for Global Food Security, School of Biological Sciences, Queen’s University Belfast, Belfast, BT9 5DL UK; 2 Food Safety Department, Teagasc Food Research Centre, Ashtown, Dublin 15, D15 KN3K Dublin, Ireland; 3 Veterinary Sciences Division, Agri-Food and Biosciences Institute, Belfast, BT43SD UK

**Keywords:** Nitrofurans, Microwave reaction, QuEChERS extraction, Phenyl-hexyl, UHPLC-MS/MS

## Abstract

A rapid analytical method was developed and validated for the analysis of eight bound nitrofurans in animal tissue, shortening laboratory turnaround times from 4 to 2 days. The majority of methodologies for nitrofuran analysis focus on the detection of only four drugs (nitrofurantoin, furazolidone, furaltadone and nitrofurazone), and is time-consuming given the 16-h overnight derivatisation step and a double liquid–liquid extraction. In this study, the narrow scope of analysis was addressed by including further four important nitrofuran drugs (nifursol, nitrofuroxazide, nifuraldezone and nitrovin). Full chromatographic separation was achieved for the metabolites of all eight nitrofurans, using phenyl-hexyl column chemistry and a rigorous optimisation of the mobile phase additives and gradient profile. The conventional, lengthy sample preparation was substantially shortened by replacing the traditional overnight water bath derivatisation with a rapid 2-h microwave-assisted reaction, followed by a modified-QuEChERS (Quick, Easy, Cheap, Effective, Rugged and Safe) extraction. This confirmatory method was fully validated in accordance with the new 2021/808/EC legislation, and was shown to perform satisfactorily when applied to incurred tissues. The decision limit (CCα) for the eight analytes ranged between 0.013 and 0.200 µg kg^−1^, showing abundant sensitivity given that the current RPA for nitrofurans is 0.5 µg kg^−1^. This innovative method can play a major role in the surveillance of the illegal use of nitrofuran drugs.

**Supplementary Information:**

The online version contains supplementary material available at 10.1007/s00216-021-03763-0.

## Introduction

**Fig. 1:**
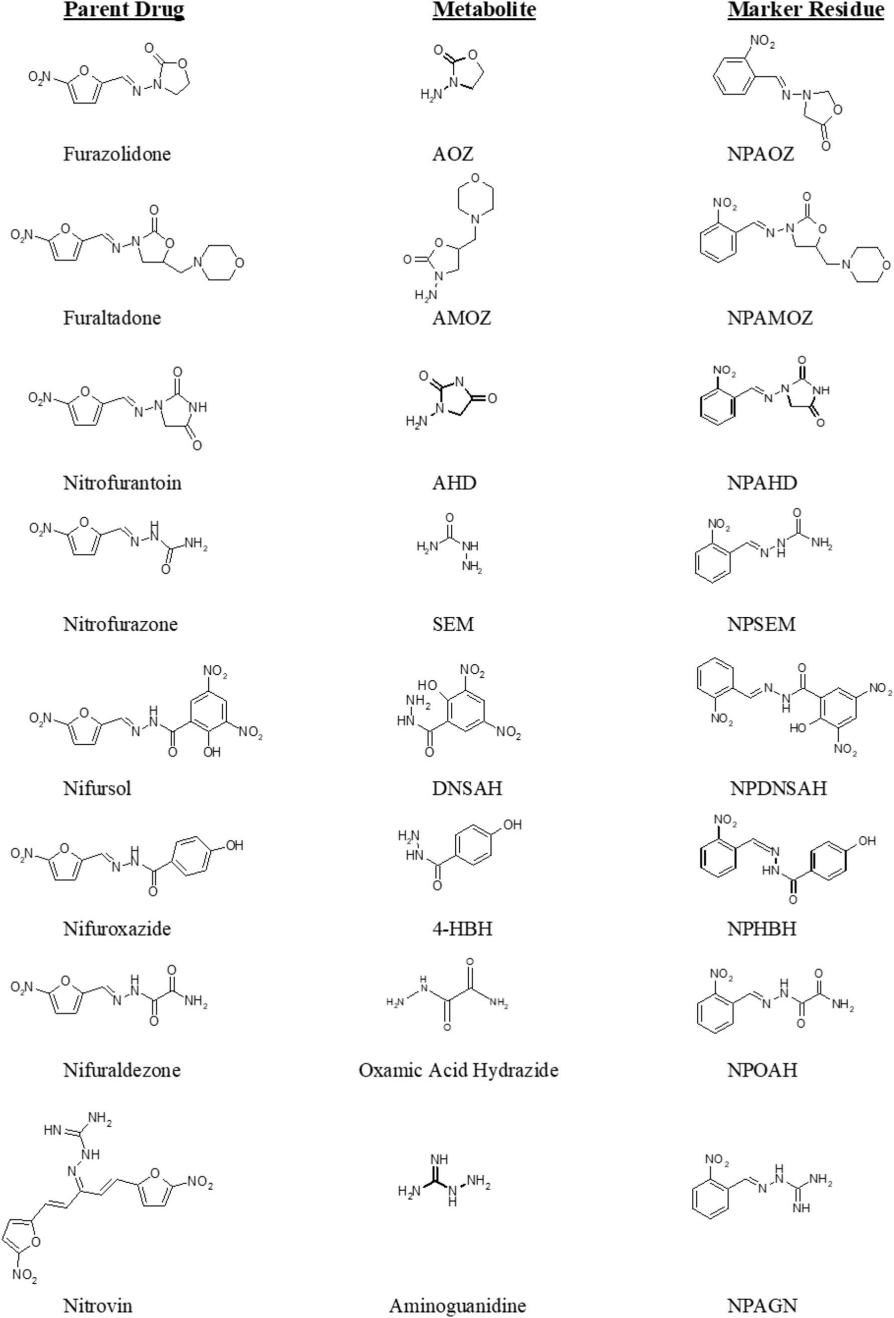
Structures of eight parent nitrofuran drugs, their respective metabolites and nitrophenyl derivative marker residues

Nitrofurans are a class of broad-spectrum antibiotics, which are characterised by their five-membered ring heterocycle structure [1] (Fig. 1). In the past, they were widely used as growth promoters and for the treatment of a range of infections and diseases [2–6], but due to concerns regarding their undesirable toxicological properties, nitrofurans are now banned from use in food-producing animals in the EU and are listed under “prohibited substances” for which an MRL (maximum residue limit) cannot be established [7]. To protect both consumer safety and food trade, the monitoring of chemical residues in food is of utmost importance [8], but due to the short half-lives of nitrofuran parent drugs in vivo, they become undetectable after a few hours and are unsuitable for monitoring purposes. However, many of the parent drugs are rapidly metabolised in vivo to form highly stable protein-bound metabolites, which are used as marker residues for analysis [9–11]. As part of an EU-funded project entitled FoodBRAND (Bound Residues and Nitrofuran Detection), a confirmatory LC–MS/MS method was developed for the analysis of four nitrofurans, namely furaltadone, furazolidone, nitrofurantoin and nitrofurazone, as their respective marker residues [11]. The analysis of nitrofurans has not changed significantly since the implementation of this FoodBRAND methodology; and so, a current priority in nitrofuran analysis is the extension of the scope of methods to include new analytes. It was only recently that a fifth bound residue, namely DNSAH (marker residue for nifursol), was added to the monitoring list in the EU. Additionally, a further drug, nitrovin, was added to the priority list of veterinary drugs in China, and the drug nifuroxazide has been identified as a nitrofuran compound that can result in bound residues in animal tissue. A further recent change was the reduction of the EU reference point for action (RPA) from 1.0 to 0.5 µg kg^−1^ [12].

The challenge in nitrofuran analysis is providing sensitive detection of residues in a short turnaround time. In order to achieve optimal sensitivity, samples must be extensively washed with organic solvent, which removes significant amounts of sample matrix and leaves only the protein remaining, leading to better sensitivity and selectivity. The methodology is time-consuming and limits sample throughput, due to this extensive washing and the overnight derivatisation approach used by most laboratories. Alternative strategies have been proposed to provide more rapid analysis of nitrofuran residues, including analysis of total residues (no washing of samples) [13, 14], analysis of bound residues using simplified washing steps [15] and methods incorporating more rapid derivatisation of analytes at higher temperatures [16–18]. The drawback with the total residue approach/simplified washing is that it generally results in less sensitive analysis and can lead to shorter chromatographic column lifetimes and more mass spectrometry instrument downtime due to source contamination problems.

The aim of this work was to develop a fast method for the analysis of the bound residues of eight nitrofuran drugs. This involved the following: (1) identification of bound marker residues and evaluation of their suitability for the nitrobenzaldehyde derivatisation method; (2) optimisation of the different components of the analytical method, including the hydrolysis/derivatisation step, the sample extraction approach and chromatographic separation; and (3) validation of the method in accordance with the new legislative guidelines set out in 2021/808/EC documentation to ensure fitness for purpose.

## Materials and methods

### Chemicals, materials and apparatus

Romil “SpS”-grade (super purity solvent) acetonitrile (MeCN) 200 far UV, methanol (MeOH) 215 and propan-2-ol (IPA) were purchased from Romil Ltd. (Cambridge, UK). Ethanol (EtOH) absolute and trisodium phosphate dodecahydrate were sourced from Merck KGaA (Darmstadt, Germany). 2-Nitrobenzaldehyde (NBA), ammonium formate puriss p.a. (puriss pro analysis) and concentrated hydrochloric acid (HCl) (37%) were supplied by Sigma-Aldrich (Dublin, Ireland). Diethyl ether was sourced from Honeywell (Riedel–de Haen; Seelze, Germany). Enviro-clean anhydrous magnesium sulphate (MgSO_4_) was obtained from United Chemical Technologies Ireland Ltd (Wexford, Ireland). Sodium chloride (NaCl) was purchased from Applichem (Darmstadt, Germany). Ultra-pure water (UPW) (18.2 MΩ cm^−1^) was generated in-house using a Millipore water purification system (Millipore, Cork, Ireland).

3-Amino-2-oxazolidinone (AOZ), 3-amino-5-morpholinomethyl-1,3-oxazolidin-2-one (AMOZ), 1-aminohydantoin (AHD), oxamic acid hydrazide (OAH), 5-dintrosalicylic hydrazide (DNSAH), 3-((2-nitrophenyl)methylene-amino-2-oxazolidinone (NPAOZ), 5-methylmorfolino-3-((2-nitrophenyl)methylene)-3-amino-2-oxazolidinone (NPAMOZ), 1-((2-nitrophenyl)methylene)-amino-2-hydantoin (NPAHD), (2-nitrophenyl)methylene-semicarbazide (NPSEM), 3,5-(2-nitrophenyl)-dinitrosalicylic acid hydrazide (NPDNSAH), 4-(2-nitrophenyl)-hydrozybenzhydrazide (NPHBH) and (2-nitrophenyl)-aminoguanidine (NPAGN) were all purchased from Witega (Berlin, Germany). Semicarbazide hydrochloride (SEM) and aminoguanidine hydrochloride (AGN) were both supplied by Sigma-Aldrich (Dublin, Ireland). 4-Hydroxybenzhydrazide (HBH) was obtained from Toronto Research Chemicals (Toronto, ON, Canada). 2-Nitrophenyl-oxamic acid hydrazide (NPOAH) was obtained via laboratory synthesis at Teagasc Food Research Centre (Ashtown, Ireland). All internal standards were purchased from Witega (Berlin, Germany), namely, 3-amino-2-oxazolidinone-d4 (AOZ-d4), 3-amino-5-morpholinomethyl-1,3-oxazolidinon-2-one-d5 (AMOZ-d5), 1-aminohydantoin-^13^C_3_ (AHD-^13^C_3_), semicarbazide-^13^C ^15^N_2_ (SEM-^13^C ^15^N_2_), 3,5-dinitrosalicylic acid hydrazide-^13^C_6_ (DNSAH-^13^C_6_), 4-hydroxybenzhydrazide (HBH-^13^C_6_), oxamic acid hydrazide-^15^N_3_ (OAH-^15^N_3_) and aminoguanidine-^13^C^15^N_4_ (AGN-^13^C^15^N_4_).

Polypropylene tubes (15 mL and 50 mL) were obtained from Sarstedt Ltd (Wexford, Ireland). A ME365 microbalance, purchased from Sartorius (Dublin, Ireland), was used for the weighing of standard material during standard preparation. An Ultra-Turrax probe blender from IKA (Staufen, Germany), a Mars 6 240/50 model microwave from CEM Microwave Technology Ireland Ltd (Dublin, Ireland), a Minimix vibrational unit (Merris Engineering Ltd., Milltown, Co. Galway, Ireland) and a TurboVap LV evaporator from Biotage (Sweden) were used during sample preparation. PTFE cross stirrer bars (8 × 20 mm) were purchased from VWR International Ltd (Dublin, Ireland).

### Preparation of standard solutions

Individual primary stock solutions of AOZ, AMOZ, AHD, SEM, DNSAH, HBH, OAH, AGN, NPAOZ, NPAMOZ, NPAHD, NPSEM, NPDNSAH, NPHBH, NPOAH, NPAGN, AOZ-d4, AMOZ-d5, AHD-^13^C_3_, SEM-^13^C ^15^N_2_, DNSAH-^13^C_6_, HBH-^13^C_6_, OAH-^15^N_3_ and AGN-^13^C^15^N_4_ were each prepared at a concentration of 50 µg mL^−1^ in MeOH. A stock mixture (MM1) of the eight metabolites, AOZ, AMOZ, AHD, SEM, DNSAH, HBH, OAH and AGN, was prepared in MeOH from the individual stock solutions (50 µg mL^−1^) to give a concentration of 1 mg L^−1^. A stock mixture (NP1) of the eight nitrophenyl derived standards, NPAOZ, NPAMOZ, NPAHD, NPSEM, NPDNSAH, NPHBH, NPOAH and NPAGN, was prepared in MeOH at a concentration of 1 mg L^−1^ (free metabolite equivalents). The volume of each nitrophenyl-derived standard to be added to the NP1 mix was determined by calculating the ratio of the nitrophenyl derivative’s molecular weight to the free analyte’s molecular weight, and multiplying this ratio by the expected volume. The NP1 solution (1 mg L^−1^) was further diluted in MeOH to a concentration of 10 µg L^−1^ (free metabolite equivalents) to make an intermediate working solution (NP2) of the nitrophenyl derived standards. A stock mixture (IS1) of the eight internal standards, AOZ-d4, AMOZ-d5, AHD-^13^C_3_, SEM-^13^C^15^N_2_, DNSAH-^13^C_6_, HBH-^13^C_6_, OAH-^15^N_3_ and AGN-^13^C^15^N_4_, was prepared in MeOH at a concentration of 1 mg L^−1^. After preparation, the primary stock standards, MM1 standard mix, IS1 internal standard mix and NP2 nitrophenyl derivative mix solutions were stored in 25-mL screw neck amber vials, sealed by polypropylene screw caps with PTFE liners, in an explosion-proof freezer at − 20 °C. The concentrated stock solutions, MM1, NP1 and IS1, were shown to be stable for a period of at least 2 years.

Three intermediate standard mixtures were prepared daily by dilution of the MM1 solution in MeOH to give concentrations of 50 µg L^−1^ (MM2), 5 µg L^−1^ (MM3) and 0.5 µg L^−1^ (MM4). Two intermediate mixtures of internal standards were also prepared daily, by dilution of the IS1 solution in MeOH to give concentrations of 50 µg L^−1^ (IS2) and 5 µg L^−1^ (IS3).

### Development of microwave-assisted reaction

#### Optimisation of microwave conditions with incurred material

The five programmable parameters on the CEM microwave for derivatisation were (1) stirring speed, (2) power, (3) temperature, (4) time taken to ramp to chosen temperature (ramp time) and (5) time held at the chosen temperature (hold time). In this research, the stirring speed was set to ‘high’, due to the size of the magnetic stirring bars, which required high speed to have sufficient power to stir a 1-g tissue sample. Following discussions with the supplier, the microwave’s power was set to an arbitrary value of 650 W. It was advised that the exact value was negligible, given that the microwave would only use the power required to reach the desired temperature and would not exceed that value. The microwave parameters were initially optimised using spiked material, but it was found that the chosen reaction of a 4 min ramp to 65 °C and a hold of 9 min was not sufficient to hydrolyse the bound residues when applied to incurred tissues.

To obtain accurate data on the efficiency of the microwave parameters on derivatisation and hydrolysis, a large quantity of incurred material was required. From a withdrawal study carried out in the early 2000s [19], our lab had access to porcine muscle incurred with a concentrated level of AOZ. This muscle tissue was homogenised in a small blender (Kenwood mini chopper) with dry ice, which was left to sublime overnight in a cold room (4 °C). This material was analysed (using the ISO17025 method with an overnight derivatisation in a water bath at 37 °C), to determine its concentration and homogeneity. For the optimisation trials, a concentration of approximately 1 µg kg^−1^ was desired. Hence, once the concentration had been determined, the homogenous porcine muscle material was diluted with blank porcine muscle and blended with dry ice, to achieve a 1 µg kg^−1^ level.

The homogenous 1 µg kg^−1^ material was used to assess various conditions, comparing the results obtained to those achieved by overnight water bath incubation at 37 °C. To begin, a larger stirrer bar (8 × 20 mm) was trialled to assess whether the samples were simply not being sufficiently agitated by the small stirrer bars (10 × 3 mm). Following this experiment, all other derivatisation reactions were carried out using the larger stirrer. For each condition, control material was fortified in triplicate at a concentration of 1 µg kg^−1^ (200 µL MM3), to act as a single point calibration curve for each set of conditions. The fortified samples in triplicate and the incurred samples in triplicate were all spiked with internal standard at a concentration of 0.5 µg kg^−1^ (six tubes for each condition). Immediately after each trial was run, the tissue sample was removed from the microwave and was neutralised by adding 1 mL trisodium phosphate buffer (0.3 M) and approximately 570 µL NaOH (1 M). Given the reaction was dependent on an acidic environment, neutralisation stopped the derivatisation, and this step was essential for limiting the influence of factors outside of the experimental design. Samples were then extracted as per the normal procedure, described in the ‘Sample preparation’ section.

#### Impact of microwave conditions on analyte stability

The impacts of various derivatisation conditions on analyte stability were assessed by spiking empty tubes at a concentration of 1 µg kg^−1^ with the nitrophenyl derivative standard mix (100 μL NP2) and derivatisating (*n* = 3) tubes using each set of parameters. The stability studies were performed across 3 days, to assess the temperatures of 65 °C, 60 °C and 55 °C. Each trial was carried out in triplicate using a 4-min ramp time to the chosen temperature, with varying hold times: (a) 9 min, (b) 20 min, (c) 30 min, (d) 40 min, (d) 50 min, (e) 60 min, (f) 90 min, (g) 2 h, (h) 3 h and (j) 4 h. One set of three tubes was also derivatised using the routine derivatisation method of an overnight incubation in a water bath at 37 °C, to be used as a point of comparison. To determine the stability of the analytes after the derivatisation reactions, the mean response (*n* = 3) for each set of conditions was compared to the mean response (*n* = 3) for the set of control tubes that did not undergo any form of derivatisation.

#### Sample preparation

Sample aliquots (1.0 ± 0.01 g) of muscle tissue were weighed into 50-mL polypropylene tubes. Samples were homogenised with a probe, before washing with water and organic solvents, ice-cold methanol, ice-cold ethanol and diethyl ether as described by Hoogenboom et al. [20]. The samples were placed in a fume hood, and the residual diethyl ether was left to evaporate overnight. Once fully evaporated, seven matrix calibrants were fortified across a range of concentrations, from 0.02 to 5.00 µg kg^−1^, with the appropriate standard mixtures (MM2, MM3, MM4) (Table 1). A 100-µL volume of the internal standard solution (IS3) was added to all calibrants, controls and test samples. All samples were allowed to stand for 15 min.

**Table 1:** Fortification of matrix calibrant samples

Concentration in sample (μg/kg)	Standard mixture MM (μL)	Internal standard mixture IS3 (μL)
0.02	40 μL MM4	100
0.04	80 μL MM4	100
0.2	40 μL MM3	100
0.5	100 μL MM3	100
1	200 μL MM3	100
2	40 μL MM2	100
5	100 μL MM2	100

A 9-mL volume of HCl (0.1 M), a 100-µL volume of NBA (100 mM) and a magnetic stirrer bar were added to each sample. The samples were derivatised at the ‘high’ stirring speed setting in a MARS 6 microwave system by ramping from room temperature to 60 °C over 4 min, followed by a 2-h hold at 60 °C. After removal from the microwave, samples were immediately neutralised, by adding 1 mL trisodium phosphate buffer (0.3 M) and approximately 570 µL NaOH (1 M). The pH of neutralised samples was checked using pH strips (satisfactory range of pH 6.5 – 7.5) and the pH was adjusted accordingly using 1 M HCl or 1 M NaOH if necessary. Neutralised samples were then subjected to a QuEChERS-based extraction, which excluded the sorbent clean-up step due to the extensive pre-washing of the samples. In total, 10 mL MeCN, a ceramic homogeniser, and 1 g NaCl were added to each tube, before being vortexed for 1 min. Approximately 4 g of MgSO_4_ was added, and samples were shaken on a Minimix vibrational unit for 5 min. The shaken samples were then centrifuged at 3500 rpm (2800 × *g*) (4 °C, 12 min). The supernatant was transferred into a 15-mL polypropylene tube and evaporated to dryness under nitrogen stream on a Turbovap at 40 °C. Recovery control samples were spiked when the MeCN had evaporated to 1–2 mL by adding 25 µL, 50 µL, 100 µL and 200 µL of NP2 to give the equivalent concentrations of 0.25 µg kg^−1^, 0.5 µg kg^−1^, 1.0 µg kg^−1^ and 2.0 µg kg^−1^, respectively. After evaporation, the dried extracts were reconstituted in 500 µL of injection solvent (5 mM ammonium formate in H_2_O: MeOH (90:10, v/v) and vortexed on a multi-vortexer for 1 min prior to filtration through 0.2 µM PTFE syringe filters. Extracts were filtered directly into autosampler vials, and a 10-µL volume was injected into the UHPLC-MS/MS system.

#### UHPLC-MS/MS analysis

Samples were analysed using an Exion UHPLC system, coupled to an AB Sciex 5500 + QTRAP mass spectrometer (Warrington, UK), equipped with a TurboV Ion Source. The UHPLC-MS/MS system was controlled by Analyst software (V1.7.1), and the results were processed by Sciex OS software (V1.4). Separation was performed on a stainless steel Agilent ZORBAX Eclipse Plus Phenyl-Hexyl RRHD analytical column (2.1 × 50 mm, particle size 1.8 µm), fitted with an in-line filter of 0.2-µm pore size.

The column was maintained at 40 °C. A binary gradient separation comprising of 5 mM ammonium formate in H_2_O: MeOH (90:10, v/v) (mobile phase A (MPA)) and 5 mM ammonium formate in H_2_O:MeOH (10:90, v/v) (mobile phase B (MPB)) was used at a flow rate of 0.6 mL min^−1^. The gradient was as follows: (1) 0.0–1.0 min: 95% A; (2) 1.0–5.0 min: linear decrease to 60% A; (3) 5.0–6.7 min: hold at 60% A (4) 6.7–6.8: linear decrease to 50% A; (5) 6.8–8.0: hold at 50% A; (6) 8.0–8.1: linear decrease to 0% A; (7) 8.1–9.5: hold at 0% A; (8) 9.5–9.6: linear increase 95% A, (9) 9.6–11.0: hold at 95% A, for a total run time of 11 min. A divert valve was used to minimise source contamination, with the solvent diverted to waste between (a) 0 and 2.1 min and (b) 8.4 and 11.0 min. Needle wash was MeOH:H_2_O (90:10, v/v). Sample temperature was maintained at 15 °C in the autosampler. Precursor and product ions were determined through manual tuning via teed infusion of each individual analyte with mobile phase. The tuning was carried out using generic source parameters, which were later optimised by assessing a range of values for each parameter, injecting three replicates at each value and choosing the optimum based on which achieved the greatest sensitivity. The mass spectrometer was operated in both positive and negative ionisation mode, with ion spray voltage set at + / − 1400 V. Source parameters were optimised and found to be 30 psi for curtain gas, 650 °C for source temperature, 8 psi for CAD gas and 70 psi for both ion source gas 1 (GS1) and ion source gas 2 (GS2). The declustering potentials (DP), collision energies (CE) and cell exit potentials (CXP) were specifically optimised for each individual transition, ramping each parameter across a range of values and selecting the optimum based on the greatest intensity observed (summarised in Table 2).

**Table 2:** UHPLC-MS/MS conditions for nitrofuran bound residues

Analyte	Measured ion	Precursor (*m/z*)	Product (*m/z*)	RT (min)	DP	CE	CXP	MRM
NPAHD	[M + H] ^+^	249.0	134.1/104.0	3.87	95/91	18/31	7/13	20
NPAHD-^13^C_3_	[M + H] ^+^	252.1	134.1	4.08	89	18	7	20
NPAOZ	[M + H] ^+^	236.1	134.1/104.2	4.00	91/87	19/31	16/13	20
NPAOZ-d4	[M + H] ^+^	240.1	134.3	4.00	90	19	7	20
NPSEM	[M + H] ^+^	209.1	192.1/166.2	3.53	80/67	16/15	11/9	20
NPSEM-^13^C^15^N_2_	[M + H] ^+^	212.1	168.1	3.53	59	16	9	20
NPAMOZ	[M + H] ^+^	335.1	291.1/262.2	4.88	80/84	18/25	15/14	20
NPAMOZ-d5	[M + H] ^+^	340.1	296.1	4.88	64	17	17	20
NPDNSAH	[M-H]^-^	374.0	226.0/182.1	5.67	93/87	34/30	7/5	30
NPDNSAH-^13^C_6_	[M-H]^-^	380.1	188.0	5.67	75	30	10	30
NPHBH	[M + H] ^+^	286.0	121.1/93.0	4.43	66/67	27/54	7/11	25
NPHBH-^13^C_6_	[M + H] ^+^	292.0	127.2	4.43	97	31	7	25
NPOAH	[M + H] ^+^	237.1	192.1/166.4	3.37	66/72	20/16	10/10	20
NPOAH-^15^N_3_	[M + H] ^+^	240.1	194.1	3.37	69	19	10	20
NPAGN	[M + H] ^+^	208.1	191.0/119.2	2.84	87/86	20/28	11/6	60
NPAGN-^13^C^15^N_4_	[M + H] ^+^	213.0	92.2	2.84	71	34	16	60

#### Method validation

Method validation was carried out in accordance with 2021/808/EC guidelines [21], to evaluate the following performance parameters: identification, selectivity, linearity, matrix effects, trueness, within-laboratory repeatability (WLr), within-laboratory reproducibility (WLR) and decision limit (CCα).

Identification was carried out through assessment of identification points, ion ratios and retention times. Selectivity was evaluated by injection of standard solutions of each individual analyte and internal standard, to ensure that there were no observed interferences when monitoring all transitions. Additionally, 40 samples were analysed along with reagent blanks to determine if any matrix interferences co-eluted with the analytes. Linearity was evaluated using matrix matched calibration curves, with a minimum of six points, prepared through fortification of negative controls over a range of concentrations (Table 1). A matrix effects evaluation was carried out, analysing 40 samples comprising 10 bovine, 10 avian, 10 ovine and 10 porcine muscle samples, and comparing the area counts obtained from standards spiked into solvent to those obtained from post-extraction spiked muscle samples.

Trueness and precision were assessed by carrying out within-laboratory repeatability (WLr) and within-laboratory reproducibility (WLR) studies using fortified negative samples, due to a lack of available certified reference materials (CRMs) for nitrofurans in our laboratory. These validation studies were assessed at 0.5, 1.0 and 1.5 times at the reference point for action (RPA) of 0.5 µg kg^−1^, which is set for only five nitrofurans but was applied to all eight nitrofurans in this work. WLr studies were performed on three different days by the same analyst. A different species was analysed on each day, whereby 24 replicates of the one sample from bovine, avian and porcine samples were analysed, respectively. In each WLr run, eight portions of the same negative tissue sample were fortified at each of the three validation levels (3 days of *n* = 8 replicates for each level). WLR studies were performed on three different days by three different analysts. In each WLR run, 32 different tissue samples were analysed, comprising eight avian, eight bovine, seven ovine, seven porcine, one cervine and one equine sample. The WLR runs were carried out at four concentration levels, such that eight different tissues were fortified at each validation level, plus a further eight different tissues were fortified at 2.0 times the RPA, for the purposes of calculating CCα values (must be calculated using data collected at three levels at, or above, the RPA [21]).

CCα values were calculated from the within-laboratory reproducibility data, as defined in 2021/808/EC. The decision limit (CCα) is defined as the limit above which it can be concluded with an error probability of α that a sample contains the analyte. CCα values for all substances were calculated using the calibration procedure for marker residues according to ISO 11843. CCα was calculated by plotting the signal against the added concentration. The corresponding concentration at the *y*-intercept plus 2.33 times the standard deviation of the within-laboratory reproducibility of the intercept equals the decision limit (*α* = 1%). If the generated CCα values were lower than the levels achievable for certain analytes, they were set to higher concentrations. All CCα values were verified in an analytical run by fortification of 32 samples at the selected CCα values, comprising eight different bovine, eight different ovine, eight different porcine and eight different avian samples.

#### Application of method to incurred tissues

The performance of the method when applied to incurred tissue was assessed by participating in a FAPAS proficiency test in May 2021. In this study, chicken muscle incurred with SEM was provided to 21 laboratories for analysis, and a *z*-score was assigned based on the concentration measured in the sample. Additionally, incurred pig and turkey muscle samples were supplied by ANSES Fougères, incurred with AHD, DNSAH, SEM, AOZ or AMOZ. These samples were originally provided as part of past EURL proficiency studies and as such, our laboratory had access to data regarding the assigned concentrations and *z*-scores allocated to the participating laboratories. This data was interpreted and used to calculate a proposed *z*-score for the levels detected by this method (the *z*-scores reported were not officially assigned by EURL).

The method was also applied in the analysis of 118 poultry products, which were purchased from five different supermarkets in Ireland as part of a sampling study. The poultry samples were composed of various species, including chicken, quail, duck and turkey, with different countries of origin. Details of each sample can be found in Online Resource 3 in the Supplementary information. Prior to analysis, any coatings such as breadcrumbs, batters or sauces were removed before homogenisation of the remaining poultry. An aliquot of 1 g of each sample was analysed as per the procedure described above.

## Results and discussion

### UHPLC-MS/MS method development

#### Mass spectrometry method development

The 2021/808/EC guidelines require a minimum of five identification points for each analyte in a confirmatory method for prohibited substances [21]. This criterion was met through chromatographic separation (one point) and by monitoring one precursor ion (one identification point) and two product ions (1.5 identification point each) for all analytes, resulting in a total of five identification points. The precursors to product ion transitions selected for NPAHD, NPAOZ, NPAMOZ, NPSEM, NPDNSAH and NPHBH were consistent with those in the literature [11, 22, 23]. The precursor to quantifier product ion transition for NPAGN was in agreement with the method developed by Kaufmann et al. [24], but both a qualifier product ion and internal standard transitions were not reported. NPOAH and its respective internal standard, which is not included in traditional methods, follows [M + H]^+^ protonated ions. The authors were not able to corroborate the transitions followed for this new analyte, as there are no existing methods that look for these compounds. NPOAH was monitored by the protonated ion *m/z* 237.1, with the *m/z* 192.1 and 166.4 product ions selected as the quantifier and qualifier ions, respectively. The *m/z* 192.1 quantifier ion is likely formed from the cleavage of the formamide (HCONH_3_) group.

#### Liquid chromatography method development

The majority of methods developed for analysing bound nitrofuran residues report the separation of the nitrophenyl ester derivatives using alkyl-bonded silica stationary phases [25], with a predominant focus on only four or fewer compounds, namely NPAHD, NPAOZ, NPAMOZ and NPSEM [16, 26, 27]. In this work, a BEH C_18_ column chemistry (2.1 × 100 mm; 1.7 µm) was initially evaluated but gave an unsatisfactory peak shape, particularly for NPAGN, NPAHD and NPDNSAH. In addition, matrix interfering peaks were observed in chromatographic traces that were not fully resolved from the analytes. Consequently, phenyl-hexyl column chemistries were evaluated because they can provide improved selectivity for compounds containing aromatic functionalities.

Seven different LC columns with phenyl chemistries were evaluated to determine which column achieved the best chromatographic separation of the eight nitrofuran analytes, assessing the performance based on three factors, namely, the resolution, the efficiency and the asymmetry factor. Resolution (*R*_S_) is a quantitative measure of how well two peaks can be differentiated in a chromatographic separation, with a *R*_S_ value ≥ 1.5 considered baseline resolution [28]. Column efficiency (N), or plate count, is a measure of the dispersion of a peak, with larger values indicating higher efficiency. Asymmetry factor is a measure of peak tailing, where a value of 1 is most desirable. The seven columns assessed were the Agilent ZORBAX Phenyl-Hexyl (1.8 µm), Halo 90 Å Phenyl-Hexyl (2.7 µm), Raptor Biphenyl (2.7 µm), YMC-Triart Phenyl (1.9 µm), Sigma Ascentis Phenyl-Hexyl (2.7 µm), Kinetex Phenyl Hexyl (2.6 µm) and Kinetex Phenyl Hexyl (5 µm). The assessment of the three factors, alongside visual assessment of the chromatographic separations (shown in Online Resource 1 in Supplementary information), indicated that the Agilent ZORBAX Phenyl-hexyl column performed best for the analysis of eight bound nitrofuran residues.

Mobile phase composition was assessed to optimise peak shape and separation. Methanol was found to be a more suitable organic modifier than MeCN because it gave increased retention and analyte separation. A range of different mobile phase additives were evaluated including acids and salts, which showed that ammonium formate gave superior peak shape. Ammonium formate was initially added to the mobile phase A only at a concentration of 10 mM, but column lifetime was quite short at approximately 1000 injections. This column degradation was exhibited by a dramatic increase in column backpressure. This blockage occurred in spite of the column being fitted with a guard 0.2 µm in line filter frit. Following consultation with suppliers, it was proposed that the column blockage was due to protein precipitation because of the salt gradient across the column. The column stability issue was subsequently resolved by reducing the ammonium formate concentration to 5 mM and including this additive in both mobile phases.

**Fig. 2:**
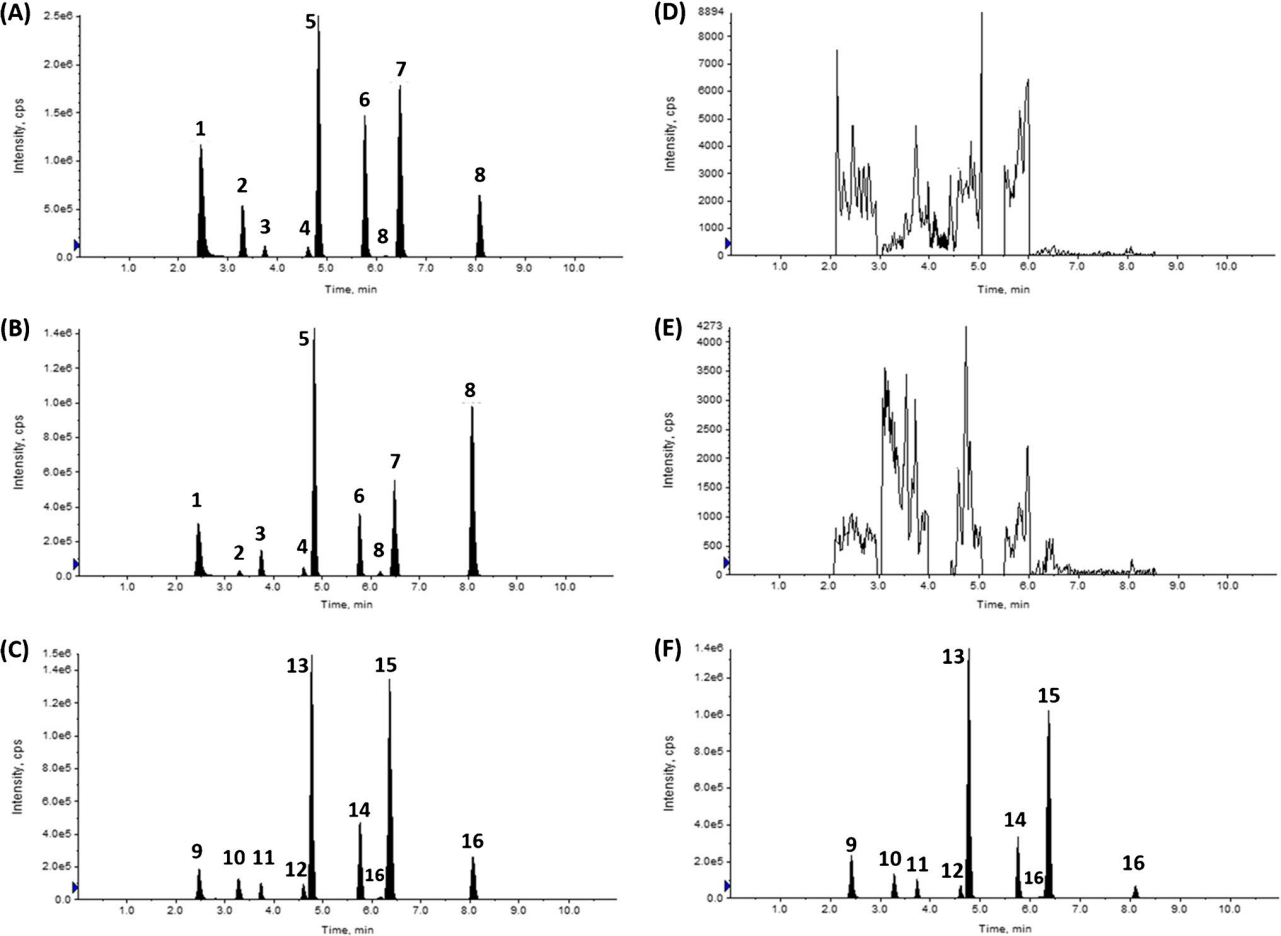
Chromatogram of a muscle sample spiked at 0.5 µg kg^−1^ for **A** quantifier transitions, **B** qualifier transitions, and **C** internal standards. Chromatogram of a blank muscle sample for **D** quantifier transitions, **E** qualifier transitions, and **F** internal standards. Chromatographic separation achieved using an Agilent ZORBAX Eclipse Plus Phenyl-Hexyl RRHD (2.1 × 50 mm; 1.8 µm) at 40 °C at a flow rate of 0.6 mL min^−1^. Analytes are labelled as follows: **1**, NPAGN; **2**, NPOAH; **3**, NPSEM; **4**, NPAHD; **5**, NPAOZ; **6**, NPHBH; **7**, NPAMOZ; **8**, NPDNSAH; **9**, NPAGN-^13^C^15^N_4_; **10**, NPOAH-^15^N_3_; **11**, NPSEM-^13^C^15^N_2_; **12**, NPAHD-^13^C_3_; **13**, NPAOZ-d4; **14**, NPHBH-^13^C_6_; **15**, NPAMOZ-d5; **16**, DNSAH-^13^C_6_

Despite the selectivity of LC–MS/MS, the development of an efficient gradient separation is critical to separate analytes from matrix interfering peaks. This is especially true for some nitrofuran analytes, such as NPAHD, which have to be measured accurately at sub-ppb concentrations. Therefore, several LC gradients were trialled to achieve the best chromatographic separation of all eight analytes, both from each other and also from any matrix-interfering peaks. The final optimal gradient selected started at a hold of 95% A for 1 min, which was essential to achieve satisfactory NPAGN peak shape. The subsequent gradient to 60% A over the course of 4 min was necessary to separate the NPAHD analyte peak from an interfering matrix peak. Any length of time shorter than 4 min caused an overlap of NPAHD with a matrix interference. The final gradient resulted in a 0.26-min separation between these peaks, which is more than sufficient for quantitation, and its robustness was verified using several column batches. The method’s chromatographic separation for the eight analytes and their respective internal standards is shown in Fig. 2, for both a negative control and a sample spiked at 0.5 µg kg^−1^.

With the optimised LC conditions, all eight analytes and eight internal standards were successfully eluted in the first 8.4 min of the gradient. After the last analyte was eluted, the gradient was held at 100% B for 1.4 min to remove matrix co-extractive from the column, before being returned to the starting gradient (95% A) for column re-equilibration, resulting in a total run time of 11 min. The LC flow was diverted to waste prior to 2.1 min, and again after 8.4 min, in order to minimise source contamination.

### Sample preparation method development

#### Optimisation of microwave-assisted derivatisation parameters

One of the main objectives of this work was to develop a microwave-assisted derivatisation reaction, which could replace the lengthy conventional approach of overnight incubation in a heated water bath, with the aim of significantly reducing the 16-h derivatisation time. Preliminary testing indicated that the optimisation trials should be carried out in matrix rather than solvent, given that particularly poor derivatisation occurred in solvent for NPAMOZ and NPAGN when compared with matrix (93% and 97% differences, respectively).

The initial optimisation work was carried out using fortified samples and the optimal microwave conditions were found to be a 4-min ramp to a 65 °C temperature, followed by a 9-min hold time, giving a total reaction time of 13 min. Upon selection of these parameters, further work was carried out to validate the performance and applicability of the method. As part of the validation work, proficiency samples were tested which highlighted that the optimised conditions were not performing at an equivalent level to the traditional overnight water bath incubation when incurred material was analysed (54% lower yield). It was proposed that the lower results achieved with the 13-min microwave derivatisation method was due to incomplete acid hydrolysis of the bound nitrofuran residues. Given the initial optimisation of the derivatisation step was carried out using only fortified tissue, it only evaluated the reaction of released bound residues with 2-NBA.

To overcome these challenges, further optimisation was carried out using porcine muscle incurred with AOZ. Larger stirrer bar sizes were tested to investigate whether the issue was simply due to insufficient agitation in the microwave. The traditional overnight incubation takes place in a shaking water bath, leading to the constant agitation of samples for 16 h, whereas the original microwave derivatisation reaction used small stirrer bars (10 × 3 mm) to stir the samples on ‘high’ speed. When the impact of using a larger stirrer bar size was assessed, the results showed an increase in yield of approximately 7%. This improvement indicated that the increased agitation of samples, and the subsequent faster rate of mass transfer, helped in the hydrolysis and derivatisation of bound nitrofuran metabolites from the protein in incurred tissues. Despite the fact that a 7% increase in yield alone was not sufficient to achieve equivalency with the traditional derivatisation approach, the larger stirrer bar size was used in all future microwave reactions.

**Fig. 3:**
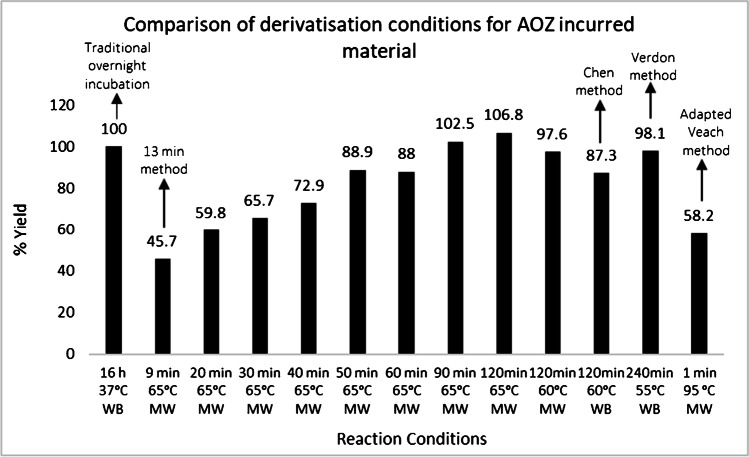
Comparison of the performance of various derivatisation conditions. % yield shown is determined by calculating the mean AOZ concentration (*n* = 3) measured with each set of conditions and expressing each value as a percentage of the AOZ concentration measured using the traditional overnight incubation at 37 °C. All microwave reactions used a ramp time of 4 min, with the exception of the 95 °C reaction which ramped over 5 min. Time shown, hold time; MW, microwave reaction; WB, heated water bath

The impact of longer hold times and varying temperatures in the microwave reaction, as well as the performance of other published derivatisation methods, was assessed (Fig. 3). Since the establishment of the original FoodBRAND method for the analysis of nitrofurans, a number of groups have reported that the use of elevated reaction temperatures can shorten the derivatisation time [17, 18, 29]. Veach et al. [18] developed a rapid method for analysing nitrofurans, using a 6-min derivatisation procedure at 95 °C. A slight drawback with this method was that it required the use of pressurised Xpress vessels for the reaction. These vessels are expensive and have to be cleaned after each analytical batch, which was considered a disadvantage and could give rise to cross-contamination. This procedure was evaluated using 50-mL polypropylene tubes but the conditions were unsuitable and caused samples to boil over. Subsequently, methods using lower reaction temperatures were investigated.

Two such protocols were reported by researchers based at the European Reference Laboratory (EURL) for antibiotic drug residues in foods of animal origin in France. In one of these methods, a reaction at 55 °C for 4 h was optimal [17], while the other protocol employed a higher temperature of 60 °C and a derivatisation time of 2 h [29]. An evaluation of these methods was carried out, showing significantly higher (*P* < 0.05) yields of bound AOZ residues using both of the EURL methods, when compared to a 16-h water bath incubation at 37 °C. A range of different reaction times were also evaluated using the original run temperature of 65 °C. The results from the optimisation indicated a derivatisation temperature between 55 and 65 °C and a run time of > 90 min gave satisfactory yield of AOZ bound residues. However, it was not possible to optimise the method thoroughly for other analytes due to a lack of incurred tissues with the different drugs.

It was reported by Johnston et al. [30] that although higher temperatures improve the release of bound residues of nitrofuran drugs, nitrophenyl derivatives for some analytes such as NPSEM can degrade at elevated temperatures. Therefore, the stability of nitrophenyl derivatives was investigated by spiking with NP derivatives just prior to derivatisation. The results from this study are shown in Online Resource 2 (Supplementary information) which compares yields for each of the eight analytes relative to pure nitrophenyl standards. On each chart, the concentration measured using the traditional overnight water bath incubation at 37 °C is depicted as a horizontal line. The stability of four derivatives, namely NPAHD, NPAMOZ, NPAOZ and NPAGN, did not appear to be affected by microwave derivatisation, and these compounds were stable under the conditions evaluated. It can be seen from the charts for the other four analytes that some degradation took place during derivatisation (as much as 40%). The trend shows that NPSEM was more stable at a reaction temperature of 37 °C than 55 – 65 °C, while NPDNSAH appeared to degrade more rapidly at 37 °C. Overall, a temperature of 60 °C was selected because it gave higher yield for the majority of analytes. Subsequently, a microwave reaction with a 4-min ramp to 60 °C and a hold of 2 h was selected as optimal.

### Method validation

#### Identification and confirmatory criteria

The 2021/808/EC guidelines outline the requirements for chromatographic separation in a confirmatory method whereby the retention time of the analyte must correspond to that of the calibration standard with a tolerance of ± 0.1 min. Furthermore, in cases where an internal standard is present, the deviation between relative retention times must be ≤ 1%. These criteria were met during all validation runs. In addition, specific performance criteria for mass spectrometric detection were met given that ion ratios were within the ± 40% tolerance for each sample tested throughout validation.

#### Linearity

Linearity was determined through visual inspection of extracted matrix-matched calibration curves, consisting of a minimum of six calibration levels, with 1/*x*^2^ weighting and linear fit, fortified across a range of concentrations in muscle (shown in Table 3). Linearity was achieved across each analyte’s calibration range, with regression coefficient values (*R*^**2**^) ≥ 0.998, while residuals were in the ± 20% range from the curves.

**Table 3:** Calibration range, mean linearity (of *n* = 8 runs), RTs, ME and RSD for nitrofuran bound residues

Analyte	Calibration range (μg kg^−1^)	*R*^2^	RT ± SD^a^ (min)	ME^b^ (RSD) (%)
NPAHD	0.02 – 5.00	0.998	4.64 ± 0.025	− 7.4 (5.1)
NPAOZ	0.02 – 5.00	0.999	4.84 ± 0.020	− 19.3 (5.8)
NPAMOZ	0.02 – 5.00	0.998	6.50 ± 0.027	− 19.0 (5.8)
NPSEM	0.20 – 5.00	0.998	3.75 ± 0.019	+ 10.2 (7.1)
NPHBH	0.02 – 5.00	0.999	5.79 ± 0.024	− 34.2 (9.2)
NPAGN	0.02 – 5.00	0.999	2.45 ± 0.023	− 44.3 (10.3)
NPOAH	0.02 – 5.00	0.999	3.30 ± 0.018	− 63.6 (19.2)
NPDNSAH	0.02 – 5.00	0.999	8.15 ± 0.046	− 30.6 (8.8)

^**a**^Standard deviation (between-runs; *n* = 8)

^b^A positive ( +) ME value indicates ion enhancement and a negative (-) ME value indicates ion suppression

#### Selectivity and matrix effects

**Fig. 4:**
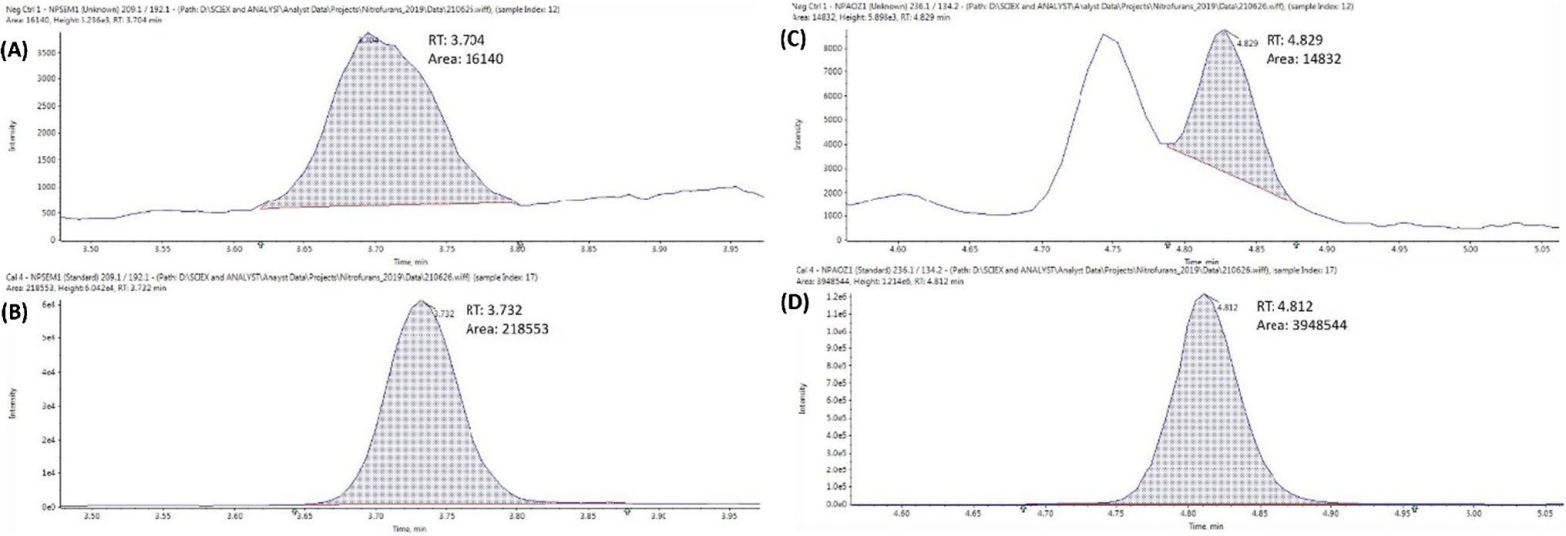
**A** Interfering peak for NPSEM observed in the negative control. **B** Analyte peak for NPSEM spiked at RPA of 0.5 µg kg^−1^. **C** Interfering peaks for NPAOZ observed in the negative control. **D** Analyte peak for NPAOZ spiked at RPA of 0.5 µg kg^−1^

The establishment of selectivity is a critical aspect of method development and was achieved by efficient chromatographic separation. To evaluate the selectivity of the method, the 2021/808/EC guidelines require that a minimum of 20 blank samples should be analysed and checked for interferences. In this work, 40 different muscle (10 bovine, 10 avian, 10 ovine and 10 porcine) samples were tested as part of a matrix effects study. A small interfering peak was observed in the quantifier (209.1 > 192.1* m/z*) and qualifier (209.1 > 166.2 *m/z*) transitions for NPSEM, in all samples tested during this evaluation, the largest of which was measured at approximately 6% of the RPA. For approximately 50% of samples tested, this peak satisfied the ion ratio criteria and as such, the CCα for NPSEM was established at a level (0.20 µg kg^−1^), whereby the interfering peak contributed to less than 10% of the CCα and did not impact on ion ratio tolerances. During chromatographic developmental work, matrix-interfering peaks were observed for four other analytes, namely NPAHD, NPAOZ, NPHBH and NPDNSAH. However, the interferences for NPAHD, NPHBH and NPDNSAH were satisfactorily separated from their respective analytes and moved outside of the MRM windows. Two matrix-interfering peaks were observed in the MRM window for NPAOZ, one of which was resolved from the NPAOZ analyte retention time (≥ 0.1 min separation) and did not affect quantification. In 25% of the samples analysed, the other matrix interfering peak was observed at the analyte’s retention time; the largest of which measured at just 0.3% of the RPA. The intensity of this matrix peak was so low that it was not considered to impact analysis and quantification. The interfering peaks for NPSEM and NPAOZ are shown in Fig. 4. No other interferences were observed at the retention times for any of the analytes and crosstalk was not evident from the eight isotopically labelled internal standards.

Matrix effects were calculated using the following equation: (*B* – *A*)/A*100, whereby *A* is the area counts obtained from standards spiked into solvent and *B* is the area counts obtained from post-extraction spiked samples (at 0.5 µg kg^−1^). In this study, a positive ME value indicated ion enhancement, whilst a negative value indicated ion suppression, showing how the matrix components either increased or decreased the analyte’s response. This study analysed 40 samples, comprising 10 bovine, 10 avian, 10 ovine and 10 porcine, to determine the extent of the matrix effects on the eight nitrofuran analytes in the method. The results of the ME evaluation are shown in Table 3, calculated as the mean ME value across the four species. Ion suppression was observed for all analytes, ranging from –7.4% (NPAHD) to –63.6% (NPOAH), with the exception of NPSEM for which + 10.2% ion enhancement was seen. Ion suppression is a more serious issue because it reduces the sensitivity of a method. However sensitivity was not an issue for the seven analytes that showed ion suppression, due to their lowest calibration level being 0.02 µg kg^−1^ (25 times lower than the RPA). Given this method employs extensive washing of tissues with organic solvents prior to extraction, one would expect low levels of matrix effects. In this work, the matrix effects observed for four analytes were ≥ 30%, which may indicate that the acid hydrolysis step to release the bound residues from the protein is also releasing peptides and creating greater matrix effects. The 2021/808/EC legislation does not supply guidelines for the C.V. tolerances when an internal standard is not present during the matrix effects study. However, the C.V. tolerance of ≤ 20% (normalised for an internal standard) was satisfied, despite no internal standard being present.

#### Trueness, precision and CCα

Trueness and precision values, resulting from within-lab repeatability (WLr) and within-lab reproducibility (WLR) experiments, are summarised in Table 4. The 2021/808/EC guidelines specify that the trueness for mass fractions ≤ 1.0 µg kg^−1^ should be in the range 50–120%. The mean trueness for all eight analytes was more than satisfactory under all WLr conditions, ranging between 99 and 102%. Validation criteria state that precision values for mass fractions of 1.0 µg kg^−1^ should be less than 23%. WLr precision (RSD_r_) across the three validation levels was acceptable, ranging from 0.6 to 3.9%. The mean trueness was satisfactory for all eight analytes under the reproducibility conditions, ranging between 99 and 105%. WLR precision (WLR_wR_) was satisfactory, with all values in the range of 0.9 – 10.7%. Overall, the method was shown to be accurate and precise for the analysis of all eight analytes. When determining CCα values using the calibration procedure calculations, CCα values ranged between 0.013 µg kg^−1^ (NPAMOZ) and 0.058 µg kg^−1^ (NPDNSAH). However, for two of the eight analytes, namely NPSEM and NPOAH, these calculations produced CCα values at a concentration lower than achievable. Based on the chromatography generated from the WLr and WLR studies, these values were not considered achievable from the perspective of satisfying both quantitative and qualitative criteria, such as acceptable ion ratios and satisfactory signal-to-noise. Hence, to ensure the CCα values for this method were reliable and represented an achievable decision limit, the CCα concentrations were set to a higher level, as shown in Table 4, and verified in an analytical run comprising bovine, avian, porcine and ovine muscle tissues.

**Table 4:** Validation results for the analysis of eight bound nitrofuran metabolites in a range of avian, bovine, ovine and porcine muscle samples

Analyte	WLr trueness (%) (RSDr) (%)			WLR trueness (%) (RSDR) (%)				Verified CCα (μg kg^−1^)
	L1	L2	L3	L1	L2	L3	L4	
NPAHD	100 (2.8)	100 (1.7)	100 (1.9)	99 (2.4)	100 (2.0)	99 (3.9)	101 (4.0)	0.030
NPAOZ	101(2.0)	100 (2.1)	100 (1.2)	100 (1.6)	100 (2.5)	99 (2.8)	99 (1.9)	0.019
NPAMOZ	100 (2.6)	100 (2.0)	100 (1.4)	101 (2.4)	100 (1.8)	100 (1.4)	101 (1.7)	0.013
NPSEM	100 (2.5)	101 (3.9)	99 (1.0)	101 (3.7)	100 (3.8)	100 (2.1)	100 (2.8)	0.200
NPHBH	101 (2.6)	101 (2.1)	100 (1.6)	100 (2.4)	99 (4.3)	100 (9.6)	98 (6.0)	0.070
NPAGN	100 (2.5)	101 (2.0)	100 (0.6)	101 (2.0)	101 (0.9)	101 (2.6)	101 (2.1)	0.017
NPOAH	100 (2.5)	100 (1.5)	100 (0.8)	101 (2.2)	100 (1.4)	100 (2.5)	100 (2.6)	0.200
NPDNSAH	101 (3.9)	102 (3.9)	101 (2.7)	99 (4.5)	101 (3.5)	105 (10.7)	100 (3.4)	0.058

#### Application of method to incurred tissues

The method presented in this study was successfully applied to a range of incurred materials. In the FAPAS proficiency study, SEM was detected and  a *z*-score of 0.0 was assigned, showing satisfactory method performance. Additional incurred turkey and pork muscle tissues, of known concentrations, were analysed and the levels measured were compared to their respective assigned concentrations (Table 5). Given that a *z*-score of − 2.0 ≥ *x* ≤ 2.0 is deemed satisfactory in a proficiency study, the six incurred muscle samples, incurred with AHD, AOZ, AMOZ, SEM or DNSAH, met the criteria for suitable method performance.

**Table 5:** Performance of method when applied to incurred muscle tissue

Sample ID	Source	Analyte detected	Species	Assigned concentration (μg kg^−1^)	Measured concentration (μg kg^−1^)	Proposed *z*-score
02,429	FAPAS	NPSEM	Chicken	2.560	2.549	0.00
15JJ-9	EURL	NPAHD	Pig	1.701	1.435	− 0.49
20QY-144	EURL	NPAOZ	Pig	0.456	0.563	+ 1.07
20QY-24	EURL	NPAMOZ	Turkey	0.294	0.313	+ 0.30
17NHD214	EURL	NPSEM	Pig	0.871	0.702	− 0.88
20QY-89	EURL	NPSEM	Pig	0.558	0.470	− 0.72
20QY-91	EURL	NPDNSAH	Turkey	0.239	0.234	− 0.09

The method was also applied to 118 poultry samples as part of a retail sampling study. All samples were found to be negative given that no nitrofuran bound residues were detected. This result would indicate that there is no misuse of nitrofuran antibiotics in poultry across several countries, including China, Thailand, Brazil and many European countries. However, caution must be applied when arriving at this conclusion, such that this selection of samples is only a snapshot representation of poultry at a certain point in time. The poultry sampling survey presented in this paper highlights the suitability and robustness of this confirmatory method, given that it was applied to a wide range of different processed and unprocessed samples. The method was fit to analyse all sample types, and no additional interferences were observed.

## Conclusions

A rapid and sensitive analytical method, incorporating a microwave-assisted derivatisation reaction and a modified QuEChERS extraction, has been developed for the confirmatory analysis of eight bound nitrofurans in animal tissue. The method has undergone extensive validation in accordance with the new 2021/808 legislation across a range of concentrations, in line with the 0.5 µg kg^−1^ RPA for nitrofurans. The traditional approach to bound nitrofuran analysis is lengthy due to the overnight derivatisation step, followed by a double liquid–liquid extraction. This work proposes an alternative rapid approach using a 2-h microwave reaction and a modified QuEChERS extraction, shortening analysis time from 4 to 2 days. Based on currently available literature, this method is the first of its kind to comprehensively detect each of the eight nitrofurans as their respective marker residues. The findings during this study highlighted the importance of applying newly developed methods to incurred materials, particularly when analysing for bound residues, to ensure fitness for purpose. Overall, through rigorous validation studies and partaking in proficiency tests, the method presented in this paper has shown satisfactory performance in the analysis of eight bound nitrofurans in meat. This method can play a major role going forward in the surveillance for the illegal use of nitrofuran drugs.

## Supplementary Information

Below is the link to the electronic supplementary material.

Supplementary file1 (DOCX 608 KB)

## Data Availability

All data generated during this study are included in this published article and its supplementary information files.
