# Supplementary material for: Development and validation of a rapid LC–MS/MS method for the confirmatory analysis of the bound residues of eight nitrofuran drugs in meat using microwave reaction
Source: Anal Bioanal Chem. 2021 Nov 23;414(3):1375–88. doi: 10.1007/s00216-021-03763-0 (PMC8724188; doi:10.1007/s00216-021-03763-0)
Supplement: Supplementary file 1 — Supplementary file1 (DOCX 608 KB) [file 216_2021_3763_MOESM1_ESM.docx]

**Online Resource 1**

**Online Resource 1** Comparison of chromatographic separation achieved using six different phenyl-based column chemistries, namely **(A)** Agilent ZORBAX Eclipse Plus Phenyl-Hexyl RRHD (2.1 × 50 mm; 1.8 µm), **(B)** Halo 90 Å Phenyl-Hexyl (2.1 × 50 mm; 2.7 µm). **(C)** YMC-Triart Phenyl (2.0 × 50 mm; 1.9 µm), **(D)** Sigma Ascentis Express Phenyl-Hexyl (2.1 × 50 mm; 2.7 µm), **(E)** Phenomenex Kinetex Phenyl Hexyl (2.1 × 50 mm; 2.6 µm) and **(F)** Phenomenex Kinetex Phenyl Hexyl (2.1 × 50 mm; 5 µm). Analytes are labelled as follows: **1:** NPAGN, **2:** NPOAH, **3:** NPSEM, **4:** NPAHD, **5:** NPAOZ, **6:** NPHBH, **7:** NPAMOZ, **8:** NPDNSAH.

**Online Resource 2**


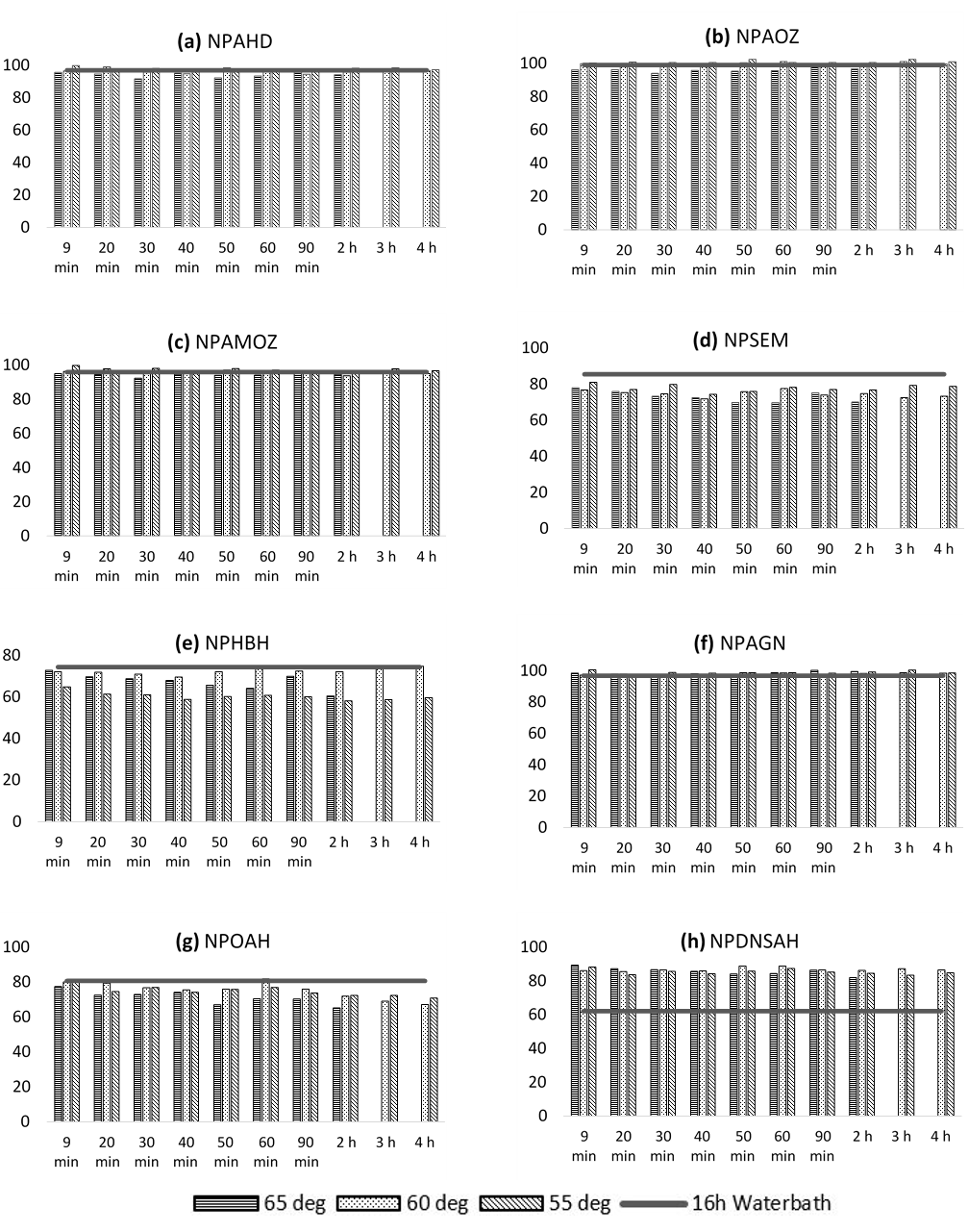


**Online Resource 2** Impact of temperature and hold time on the response of **(a)** NPAHD, **(b)** NPAOZ, **(c)** NPAMOZ, **(d)** NPSEM, **(e)** NPHBH, **(f)** NPAGN, **(g)** NPOAH and **(h)** NPDNSAH, relative to the response obtained when no derivatisation was applied.

**Online Resource 3:** Poultry samples purchased and analysed as part of the supermarket sampling study

| **Sample** | **Product Type** | **Supermarket** | **Meat Origin** |
| --- | --- | --- | --- |
| 1 | Whole quail | A | France |
| 2 | Duck breast fillets | A | France |
| 3 | Breaded chicken goujons | A | UK and EU |
| 4 | Chicken breast fillets | A | Ireland |
| 5 | Breaded chicken fillets | A | EU |
| 6 | Chicken breast fillets | A | Ireland |
| 7 | Chicken breast fillets | A | Ireland |
| 8 | Crispy shredded chicken | A | China |
| 9 | Chicken breast fillets | A | Ireland |
| 10 | Breaded chicken goujons | A | Northern Ireland |
| 11 | Crispy shredded chicken | A | China |
| 12 | Corn fed chicken breast fillets | A | Ireland |
| 13 | Battered chicken balls | A | China |
| 14 | Breaded chicken nuggets | A | Northern Ireland |
| 15 | Battered chicken goujons | A | EU, Brazil and Ukraine |
| 16 | Chicken breast fillets | A | Ireland |
| 17 | Chicken breast fillets | A | Ireland |
| 18 | Chicken breast fillets | A | Ireland |
| 19 | Chicken burgers | A | UK* |
| 20 | Chicken dippers | A | UK* |
| 21 | Breaded chicken goujons | A | Poland |
| 22 | Chicken tikka masala | A | Ireland* |
| 23 | Chicken tikka masala | A | Ireland* |
| 24 | Breaded chicken mini fillets | A | Ireland |
| 25 | Turkey breast mince | A | Italy |
| 26 | Chicken spring rolls | A | Thailand |
| 27 | Chicken breast fillets skin on | A | Ireland |
| 28 | Chicken breast fillets | A | Ireland |
| 29 | Duck spring rolls | A | Thailand and UK |
| 30 | Roast chicken slices | B | UK |
| 31 | Chicken breast fillets | B | UK |
| 32 | Katsu chicken tenders | B | UK |
| 33 | Chicken steaks | B | UK |
| 34 | Chicken breast fillets | B | UK |
| 35 | Sliced roast chicken | B | UK |
| 36 | Mini chicken breast fillets | B | UK |
| 37 | Chicken breast fillets | B | UK |
| 38 | Turkey breast mince | B | UK |
| 39 | Duck breast | B | UK |
| 40 | Chicken dippers in batter | C | EU |
| 41 | Chicken fillet burgers | C | EU and non EU |
| 42 | Breaded chicken burgers | C | EU and non EU |
| 43 | Curried chicken balls | C | EU and non EU |
| 44 | Breaded chicken breast fillets | C | EU and non EU |
| 45 | Chicken steaks in breadcrumbs | C | EU |
| 46 | Shredded chicken | C | EU and non EU |
| 47 | Chicken breast fillets | C | Poland |
| 48 | Shredded chicken in batter | C | EU and non EU |
| 49 | Chicken goujons in breadcrumbs | C | EU and non EU |
| 50 | Breaded chicken mini fillets | C | Ireland |
| 51 | Chicken fillet goujons | C | EU |
| 52 | Chicken skewers with glaze | C | Poland |
| 53 | Chicken breast fillets | C | Ireland |
| 54 | Chicken thigh fillets | C | Ireland |
| 55 | Roasted chicken breast pieces | C | EU |
| 56 | Free range chicken breast fillets | C | Ireland |
| 57 | Duck breast fillets | C | Ireland |
| 58 | Turkey breast steaks | C | Ireland |
| 59 | Turkey burger | C | Ireland |
| 60 | Carved Irish chicken | C | Ireland |
| 61 | Chicken breast fillet chunks | D | Thailand |
| 62 | Spiced chicken breast strips | D | Thailand |
| 63 | Crispy chicken breast fillet strips | D | Thailand |
| 64 | Southern fried chicken | D | Poland |
| 65 | Spiced chicken breast chunks | D | Thailand |
| 66 | Breaded chicken steaks | D | Thailand |
| 67 | Chicken wings | D | Poland |
| 68 | Sliced chicken breast | D | Thailand |
| 69 | Chicken mini breast fillets | D | Ukraine |
| 70 | Chicken tikka strips | D | Thailand |
| 71 | Crispy chicken | D | Germany* |
| 72 | Chicken breast slices | D | Brazil |
| 73 | Chicken breast slices | D | Thailand and Brazil |
| 74 | Turkey breast slices | D | UK, EU, Thailand and Brazil |
| 75 | Crispy chicken dippers | D | EU |
| 76 | Breaded chicken breast fillet strips | D | Thailand |
| 77 | Crispy shredded chicken | D | China |
| 78 | BBQ chicken wings | D | Poland |
| 79 | Diced chicken breast | D | Poland |
| 80 | Sweet chilli chicken mini fillets | D | Thailand |
| 81 | BBQ chicken skewers | D | EU |
| 82 | Tandoori chicken skewers | D | Thailand |
| 83 | Chicken breast slices | D | UK, EU, Thailand and Brazil |
| 84 | BBQ chargrilled chicken | D | Thailand |
| 85 | Chilli and lime chicken breast | D | Thailand |
| 86 | Crunchy chicken goujons | D | EU |
| 87 | Breaded chicken breast nuggets | D | EU |
| 88 | Breaded chicken breast chunks | D | Thailand |
| 89 | Spiced chicken breast fillets | D | Thailand |
| 90 | Chicken tikka masala | D | Ireland* |
| 91 | Crispy chicken breast fillets | D | Thailand |
| 92 | Breaded chicken breast burgers | D | EU |
| 93 | Boneless chicken box | D | Poland |
| 94 | Chicken breast fillets | D | Poland |
| 95 | Chicken breast fillets | E | Ireland |
| 96 | Southern fried chicken in breadcrumbs | E | UK* |
| 97 | Crispy chicken chunks | E | Poland |
| 98 | Chicken chargrills | E | Poland |
| 99 | Southern fried chicken steaks | E | Poland* |
| 100 | Southern fried chicken fillets | E | Poland* |
| 101 | Chicken wings | E | EU |
| 102 | Crispy chicken dippers | E | Poland |
| 103 | Breaded chicken goujons | E | EU and non EU |
| 104 | Breaded chicken burgers | E | EU |
| 105 | Duck breast fillets | E | Northern Ireland |
| 106 | Turkey breast steaks | E | Ireland |
| 107 | Chicken breast fillets | E | Ireland |
| 108 | Chicken breast fillets | E | Ireland |
| 109 | Chicken breast chunks | E | EU |
| 110 | Turkey burger | E | Ireland |
| 111 | Battered chicken | E | UK* |
| 112 | Breaded chicken steaks | E | EU |
| 113 | Chicken breast fillets | E | Ireland |
| 114 | Cajun chicken breast | E | EU |
| 115 | Cajun chicken breast carved | E | Ireland |
| 116 | Wafer thin smoked chicken breast | E | Brazil |
| 117 | Roast chicken breast carved | E | Ireland |
| 118 | Chicken breast fillets | E | Poland |

*origin not stated explicitly on packaging
